# Effects of Surface Charges on Dental Implants: Past, Present, and Future

**DOI:** 10.1155/2012/381535

**Published:** 2012-10-08

**Authors:** Cecilia Yan Guo, Jukka Pekka Matinlinna, Alexander Tin Hong Tang

**Affiliations:** Department of Dental Materials Science, Faculty of Dentistry, University of Hong Kong, 34 Hospital Road, Sai Ying Pun, Hong Kong

## Abstract

Osseointegration is a major factor influencing the success of dental implantation. To achieve rapid and strong, durable osseointegration, biomaterial researchers have investigated various surface treatment methods for dental subgingival titanium (Ti) implants. This paper focuses on surface-charge modification on the surface of titanium dental implants, which is a relatively new and very promising methodology for improving the implants' osseointegration properties. We give an overview on both theoretical explanations on how surface-charge affects the implants' osseointegration, as well as a potential surface charge modification method using sandblasting. Additionally, we discuss insights on the important factors affecting effectiveness of surface-charge modification methods and point out several interesting directions for future investigations on this topic.

## 1. Introduction

A major factor that determines the success of dental implantation is *osseointegration*, which is the stable anchorage of an implant in living bone achieved by direct bone-to-implant contacts [1, 2]. Osseointegration derives from the Greek *osteon *(*bone*) and the Latin verb *integrare* (to make whole). The term refers to the direct structural and functional connection between living bone tissues and the surface of a load-bearing dental subgingival implant. Per-Ingvar Brånemark (b. 1929), a Swedish orthopaedic surgeon and research professor acknowledged as the “father” of modern dental implantology, proposed that titanium (Ti) implants integrate such that the bone is laid very close to the implant without any intervening connective tissue. It was shown that the titanium dioxide, TiO_2_, layer permanently fuses with the bone, as Brånemark et al. showed in 1950s [3].

High-quality osseointegration stand for an accelerated healing process, high stability, and durability of the dental implant. This paper focuses on dental implants made of titanium and its alloys, which are commonly used due to their superior mechanical and biological properties. Using current materials and techniques, a titanium dental implant requires several months to osseointegrate with its adjacent bone. Moreover, the osseointegration is incomplete: analysis of retrieved titanium implants shows that the bone-to-implant attachment is far from perfect; in particular, the percentage of bone-to-implant contact area averages 70%–80%, with a minimum of 60%, even for successful implants that had lasted for up to 17 years [4]. Therefore, much room remains for the improvement of the surface quality of a titanium dental implant, in terms of the rate and strength of its osseointegration.

In light of this goal, it is crucial to understand the interactions between the host bones and the titanium implant in a living body which occurs mostly in the bone-implant interface. Both *in vitro* and *in vivo* studies showed that such interactions depend mainly upon the implant's surface characteristics [5]. Major aspects of the implant's surface characteristics include, but not limited to, surface morphology, surface chemistry, and surface energy, which significantly affect the initial bone cells' response to the implant at the bone-implant interphase [6].

Based on this theory, considerable work has been done to investigate various surface modification methods to improve the osseointegration of a titanium dental implant, such as surface-roughening (e.g., sandblasting and/or acid-etching) and coating, for example, with hydroxyapatite (HA), Ca_10_(PO_4_)_6_(OH)_2_, to improve the implant's bioactivity [5]. However, most existing methods incur some drawbacks. For instance, surface roughening methods often lead to increased soft-tissue growth onto the bone-implant interface [5], which negatively affects the contact between the implant and its host bone. On the other hand, the HA coating layer tends to disintegrate under certain circumstances, which causes cracks on the implant's surface [5]. Hence, researchers are still searching for new surface-treatment methods that avoid the above drawbacks. For instance, the use of direct silanization of polished Ti has been studied and proposed as a coating method [7].

The surface energy of a biomaterial is determined by the material's surface-charge density and the net polarity of the charge. Compared to an electrically neutral surface, a surface with net positive or negative charge may be more hydrophilic [8]. The surface-charge of a dental implant is known to be a key factor to guild bone cells adhesion and early stage bone mineralization in the bone-implant interface. Thus, surface-charge modification seems to be a promising new direction for improving the osseointegration of a titanium dental implant. Although surface-charge modification is a relatively new methodology, it has been rapidly gaining research attention in recent years. The main challenge, however, lies in effective modification of the surface-charge of the dental implant material. The main objective, hence, is to develop effective and practical techniques that create a long-lasting electric field on the implant's surface, in order to promote the implant's osseointegration without incurring the drawbacks of existing surface-treatment methods.

### 1.1. Titanium Surface: Oxides

Titanium (Ti) is the most widely used metallic material for dental subgingival implants, due to its invaluable and outstanding biomedical and biomechanical properties. These are its availability, high biocompatibility, high strength and stiffness and, relatively low density. More importantly, titanium implants are known to osseointegrate with living bone tissues. Ti is recognized for its high strength-to-weight ratio. Titanium is a strong metal with low density, and especially in oxygen-free circumstances it is also quite ductile. Ti is lustrous, metallic-white in color and it is paramagnetic, having fairly low thermal and electrical conductivity [9]. Ti is also a material of choice in prosthetic dentistry and Ti resin bonding is promoted using silica-coating methods [10].

Although the so-called commercially pure Ti has acceptable mechanical properties and has been used for orthopaedic and dental subgingival implants, for most applications titanium is alloyed with small amounts of Al and V, typically 6 wt-% and 4 wt-%, respectively. Such Ti alloy is Ti-6Al-4V (a.k.a Ti6Al4V or Ti 6–4), is the most commonly used Ti-alloy. It has a chemical composition of 6% Al, 4% V, ≤0.25% Fe (maximum), ≤0.2% O_2_, and the balance Ti. Commercially pure (c.p. Ti) is available in four grades where the oxygen content varies between 18 wt-% and 0.40 wt-% and Fe content between 0.20 wt-% and 0.50 wt-%. The apparently slight concentration differences have, however, a substantial effect on the physical and mechanical properties of c.p. Ti. At RT c.p. Ti has a hexagonal close-packed (h.c.p.) crystal lattice and is called the *α*-Ti (*so-called α*-phase). On heating, an allotropic phase transformation occurs: at 883°C, it forms a body-centered cubic (b.c.c.) lattice, labeled as *β*-phase. Ti is a reactive metal: in air and aqueous electrolytes, it forms spontaneously a dense oxide film at its surface. 

 Ti is a dimorphic metal: the *α*-form has a hexagonal structure below 882.5°C, while the *β*-form stays body-centered cubic above 882.5°C. Ti is brittle when cold, and malleable when hot, however, it can be ductile only when it is free of oxygen. On the other hand, traces of nitrogen or oxygen increase its strength. It is attacked by acids only on heating, and nitric acid, HNO_3_, oxidized Ti to TiO_2_. Melting Ti is cumbersome because at 800°C it combines with nitrogen which sets high requirements for casting Ti—a protective atmosphere is vital. Ti forms alloys with Al, Cr, Co, Cu, Fe, V, Fe, Ni and Sn [11]. 

Titanium is highly biocompatible, as a result of low-toxicity and a low rate of ion release from its surface non-toxic, and it is not rejected by the body [5]. Such properties are unanimously understood to be the consequence of an inert surface oxide film. When pure titanium or its alloys are exposed to air, a layer of titanium dioxide, TiO_2_, with a thickness of approximately 2–5 nm can often be formed in a few seconds. This thin film also protects the titanium materials, making the latter highly resistant to corrosion. TiO_2_ is insoluble in water and dilute acids but slowly dissolves in concentrated sulphuric acid. Several phases containing between 63.6–65.5 atom-% of oxygen have been indentified: these Ti oxides are of formulae TiO_1.752_ to TiO_1.902_. Titanium (III) oxide, Ti_2_O_3_, behaves as a basic oxide, and is prepared by heating TiO_2_ with carbon. Ti_2_O_3_ is a violet powder. Interestingly, Ti oxide with the valence +2, TiO, shows marked nonstoichiometry in its composition. At elevated temperatures, at around 1400°C, TiO has a defect crystal lattice over the composition range TiO_0.64_ to TiO_1.27_ and electrical neutrality is preserved in the crystal by changes in the charges on the Ti ions. Also oxides, such as Ti_3_O_5_ and Ti_2_O, have been detected and identified in special circumstances at elevated temperatures [9, 12]. 

## 2. Surface-Charge Modification for Titanium Dental Implants

### 2.1. Surface-Charge and Apatite-Layer Formation

A special group of biomaterials, including bioactive glass and glass-ceramics, have the ability to form direct bonding with bone. When such materials are inserted into living body, an intermediate biologically active bone-like apatite layer starts to form in the material-bone interface, through which the material can bond to bone [13]. Several previous studies conjecture that the formation this apatite layer on the implant's surface is a prerequisite for the implant to bond to living bone in a biological environment [14, 15]. Metals, including titanium and its alloys, cannot directly bond effectively to living bone. In order to build such bonds, various methods have been proposed to coat ceramic materials onto titanium dental implants, which help to form a biologically active bone-like apatite layer [16]. Bioactive retention can be achieved in cases where the implant is coated with bioactive materials such as hydroxyapatite. These bioactive materials stimulate bone formation leading to a physicochemical bond: the implant is anchylosed with the bone. However, as we mentioned before, the coating layer (e.g., HA) may easily peel off from its underlying titanium alloy. An alternative approach overcomes this problem by enabling titanium materials themselves to form a bone-bonding layer. Using TiO_2_ gel, Li et al. successfully induced bone-like apatite formation on titanium-based material in simulated body fluid [13]. This result shows that it is possible for titanium and its alloys to form an apatite layer though appropriate treatments.

It has also been shown that NaOH-etched and subsequently heat-treated titanium possesses the ability to directly form an apatite layer, which has been applied to artificial hip joints, and clinically used in Japan since 2007 [17]. This phenomenon is explained by the electrostatic interactions of sodium titanate, Na_2_Ti_3_O_7_, on the titanium material's surface with ions in the living body [16, 18]. The above treatment produces a negatively charged sodium titanate layer on the surface of the titanium material, which attracts positively charged Ca^2+^ ions. Ca^2+^ ions exhibit higher binding affinity compared to other cations such as K^+^, Na^+^, and Mg^2+^; consequently, Ca^2+^ are predominantly absorbed on a negatively charged biomaterial surface in a biological environment. After Ca^2+^ ions accumulate on the biomaterial's surface, the surface becomes positively charged; hence, the surface starts to attract negatively charged phosphate ions, which react with the Ca^2+^ ions to form a calcium phosphate (i.e., a type of apatite) layer [17]. This calcium phosphate layer takes an amorphous structure after its formation, and it subsequently transforms into more stable crystalline apatite.

Ever since the invention of surface-treatment methods for inducing the apatite-forming ability of titanium materials, it is believed that a negatively charged surface is essential to obtain a bioactive material with good osseointegration properties [19]. A large number of research papers have emphasized the importance of surface-charge in the formation of the apatite layer, as well as in the surface interactions between the titanium material and the biologic environment [13, 20–24]. Li et al. illustrate that a successful apatite inducer for titanium implants could be a material which has and/or develops both negative surface-charge and abundant OH^−^ groups in physiologically related fluid; such materials can thus be considered as candidates to serve for bone-bonding materials [13].

While it is widely agreed that negative surface-charge is more effective for promoting bone-implant interaction of titanium dental implants, some researchers hold the view that positive surface-charge may also be of help. For instance, it is reported that a positively charged titanium implant can develop a bone-like apatite layer [25].

### 2.2. Surface-Charge and Cell Reactions

The osseointegration of a dental implant material depends upon the cell reactions of the material, especially cell adhesion onto its surface. Cell attachment, adhesion, and spreading are the first phase of the interactions between the host cells and the implant. These reactions affect the cells' capacity to stay and proliferate on the implant's surface, and subsequently generate bone tissues surrounding the implant [26]. Cell-implant interactions depend upon the implant's surface topography, chemistry and surface energy. These properties do not only determine the adhesion of cells, but also the orientation of adsorbed molecules [8]. As described in Section 2.1, the surface energy of a material is related to the material's surface-charge. Thus, previous research has investigated the influence of the effect of implant's surface-charge on the cell reactions to the implant. It was found that on a negatively charged biomaterial surface, cells proliferate more actively; meanwhile, multiple layers of cells and enlarged colonies of osteoblast-like cells were also observed [27]. In contrast, cell adhesion and proliferation on positively charged biomaterial were found to be subdued [27].

When a biomaterial is inserted into living body, it absorbs proteins before cells adhere to its surface [28, 29]. Once attached on the material's surface, proteins can mediate cell-implant interactions [28, 29]. Cells, such as osteoblasts and fibroblasts, mainly interact with the adsorbed proteins, rather than with the implant material itself. For such cells, the implant's surface-charge influences their reactions to the implant, by affecting the type and amount of proteins attached on its surface [30].

In a biological environment, all chemical substances surrounding an electrically neutral implant, including organic and inorganic ions, proteins, ionic groups, and amino acids, have equal opportunity to contact and accumulate on implant's surface. On the other hand, a charged implant surface can induce electrical attraction or repulsion between the implant's surface and the surrounding chemical species, depending on their polarity. For example, as explained in Section 2.1, Ca^2+^ ions have superior binding affinity to a negatively charged biomaterial surface and accumulate on them [31]. Besides the effect on crystal nucleation, another significant role of Ca^2+^ is to attract cell-adhesion proteins (e.g., integrins, fibronectin, and osteonectin), which are characterized by their capacity to interact with a specific ligand [27]. These proteins significantly affect the attachment, adhesion, and spreading of osteoblasts, the cells that form bony tissues [26]. Consequently, osteoblasts attach and proliferate on a matrix grown on the bone-like apatite layer formed with Ca^2+^ ions [27], which may result in faster and stronger bone-to-implant bonding. In contrast, a positively charged implant surface attracts anionic groups which act as antiadhesive molecules, which negatively affect osteoblast adhesion [27].

Titanium naturally has a dense layer of TiO_2_ of several nanometers thick on its surface. In a biological environment (typical with pH = 7.4), the surface-charge of this TiO_2_ layer appears to be only slightly negative. Hence, researchers have devoted efforts to create a long-lasting, negative electric field on the titanium dental implant's surface.

### 2.3. Sandblasting and Titanium Surface-Charge

Sandblasting is a simple and commonly used surface-treatment method for titanium dental implants, and it has been shown to accelerate osteoblast attachment in a biological environment [26, 32], thereby enhancing the osseointegration of the treated titanium implants. Until recently, these desirable effects of sandblasting have been exclusively attributed to its roughening effect on the implant's surface. Guo et al.'s experiment reveals that current sandblasting techniques also generate a small amount of negative electric charge on the titanium material's surface [33]. Because negative surface-charge is commonly believed to promote osseointegration of a titanium material, this experiment suggests that sandblasting's favorable effects may, at least, be partially explained by this electrical phenomenon.

In Guo et al.'s experiment [33], Al_2_O_3_ grits were blasted using compressed air onto different groups of titanium plates. After the sandblasting, an electrostatic meter were place adjacent to each titanium plate to measure for static voltage on titanium plants, which is proportional to the amount of electrical charge on the plate's surface. The results show that immediately after sandblasting, the titanium plate exhibits negative static voltage, meaning that negative charge is present on the plate. The value of the static voltage (i.e., the amount of charge) is affected by the sandblasting duration, as well as environmental factors, such as atmospheric humidity.

The static charge generated by sandblasting, however, decays with time. In this study, immediately after sandblasting finishes, the static voltage of a titanium plate quickly decreases [33]. This voltage drop gradually slows down, until reaching a stable value, which is often a fraction of the initial voltage obtained by sandblasting. These findings suggest that there is abundant room for refinement of sandblasting's effect on the generation of surface-charge on titanium implants, which may promise significant improvement of the osseointegration properties of sandblasted titanium dental implants.

### 2.4. Future Research Work to Improve Surface-Charge Generation

As a simple and economical technique to generate surface-charge s on titanium surface, it may be of industrial interest to look into the research issues of sandblast-induced surface-charge. Although exploratory work has been done for enhancing the osseointegration of dental implants through sandblast-induced surface-charge modification, there remain several interesting directions of work that are worth of further investigations. On the theoretical side, better understanding of the underlying mechanism for charge generation during sandblasting is important. To be more precise, it is crucial to understand where the electrical charge comes from, why and how the charge remains on the titanium surface, how much charges are needed, and the factors that affect the amount of charge generated during the sandblasting process. Such insights help us design and develop better techniques for sandblast-induced surface-charge generation. 

On the practical side, since a typical dental implant takes the complex shape of a screw, it is of major interest to study the distribution of charge on the dental implant's surface. This information is critical for targeted charge strengthening on a screw. Another critical task is to retain the negative charge on the implant's surface, in order to further improve its osseointegration property. As reviewed in previous sections, the electrical charge that remains on the sandblasted titanium materials' surface gradually dispersed into the atmosphere. Therefore, two possible methodologies for the retention of the negative charge on the implant's surface are (i) generation of a higher amount of sufficient initial negative charges to allow for natural decay of charges up to the expiration date of the implant product, and/or (ii) to retain the surface-charge of the implant after an initial charging step. These approaches must, at the same time, be compatible to the therapeutic level of charges present at the moment of implant insertion to the patient.

Regarding the specific technique of generating charge through sandblasting-induced surface-charge, yet another industrial interesting direction for further study is to investigate the major parameters for the sandblasting that influence the generation of surface-charge for on the titanium dental implant. Example of such factors includes the materials used in blasting the implant, the size of the grits, and the blasting speed. Furthermore, it is interesting to study whether acid etching, which is commonly applied together with sandblasting, can help improving the surface-charge of the titanium implant.

## 3. Conclusion

This paper has summarized our current knowledge about the role of surface-charge on the osseointegration properties of titanium dental implants, and reviewed the state-of-the-art surface-charge modification methods for such implants. Specifically, we have described two known mechanisms for surface-charge to affect the implants' osseointegration, that is, by forming an apatite layer, and by attracting certain types of proteins with desirable reactions from bone-forming cells. Regarding surface-charge modification methods, we have presented a recently proposed original work on the modification of the surface-charge of titanium materials through sandblasting, and pointed out several important directions on this topic for further investigations to enable this technique practically. 

## References

[1] Albrektsson T, Johansson C (2001). Osteoinduction, osteoconduction and osseointegration. *European Spine Journal*.

[2] Kakuta S, Miyaoka K, Fujimori S, Lee WS, Miyazaki T, Nagumo M (2000). Proliferation and differentiation of bone marrow cells on titanium plates treated with a wire-type electrical discharge machine. *The Journal of oral implantology*.

[3] Brånemark PI, Zarb GA, Albreksson T (1986). Tissue-integrated prostheses. osseointegration in clinical dentistry. *Plastic & Reconstructive Surgery*.

[4] Albrektsson T, Eriksson AR, Friberg B (1993). Histologic investigations on 33 retrieved nobelpharma implants. *Clinical Materials*.

[5] Guo CY, Tang ATH, Matinlinna JP (2012). Insights into surface treatment methods of titanium dental implants. *Journal of Adhesion Science & Technology*.

[6] Amaral IF, Cordeiro AL, Sampaio P, Barbosa MA (2007). Attachment, spreading and short-term proliferation of human osteoblastic cells cultured on chitosan films with different degrees of acetylation. *Journal of Biomaterials Science, Polymer Edition*.

[7] Matinlinna JP, Areva S, Lassila LVJ, Vallittu PK (2004). Characterization of siloxane films on titanium substrate derived front three aminosilanes. *Surface and Interface Analysis*.

[8] Boyan BD, Hummert TW, Dean DD, Schwartz Z (1996). Role of material surfaces in regulating bone and cartilage cell response. *Biomaterials*.

[9] Bell CF, Lott KAK (1972). *Modern Approach To Inorganic Chemistry*.

[10] Matinlinna JP, Vallittu PK (2007). Silane based concepts on bonding resin composite to metals. *Journal of Contemporary Dental Practice*.

[11] Heslop RB, Robinson PL (1967). *Inorganic Chemistry*.

[12] Clark RJH (1968). *The Chemistry of Titanium and Vanadium*.

[13] Li P, Ohtsuki C, Kokubo T, Nakanishi K, Soga N, De Groot K (1994). The role of hydrated silica, titania, and alumina in inducing apatite on implants. *Journal of Biomedical Materials Research*.

[14] Hench LL (1991). Bioceramics: from concept to clinic. *Journal of the American Ceramic Society*.

[15] Kokubo T (1991). Bioactive glass ceramics: properties and applications. *Biomaterials*.

[16] Kim HM, Miyaji F, Kokubo T, Nakamura T (1996). Preparation of bioactive Ti and its alloys via simple chemical surface treatment. *Journal of Biomedical Materials Research*.

[17] Pattanayak DK, Kawai T, Matsushita T, Takadama H, Nakamura T, Kokubo T (2009). Effect of HCl concentrations on apatite-forming ability of NaOH-HCl- and heat-treated titanium metal. *Journal of Materials Science*.

[18] Nishiguchi S, Fujibayashi S, Kim HM, Kokubo T, Nakamura T (2003). Biology of alkali- and heat-treated titanium implants. *Journal of Biomedical Materials Research A*.

[19] Kokubo T, Pattanayak DK, Yamaguchi S (2010). Positively charged bioactive Ti metal prepared by simple chemical and heat treatments. *Journal of the Royal Society Interface*.

[20] Ducheyne P, Bianco P, Radin S, Schepers E, Ducheyne P, Kokubo T, van Blitterswijk CA (1993). Bioactive materials: mechanisms and bioengineering considerations. *Bone Bioactive Biomaterials*.

[21] Ducheyne P, Cuckler JM (1992). Bioactive ceramic prosthetic coatings. *Clinical Orthopaedics and Related Research*.

[22] Hench LL, Ethridge EC (1982). *Biomaterials: An Interfacial Approach*.

[23] Kokubo T, Ducheyne P, Kokubo T, van Blitterswijk CA (1992). Bioactivity of glasses and glass ceramics. *Bone-Bonding Materials*.

[24] Li P, Zhang F (1990). The electrochemistry of a glass surface and its application to bioactive glass in solution. *Journal of Non-Crystalline Solids*.

[25] Puleo DA, Nanci A (1999). Understanding and controlling the bone-implant interface. *Biomaterials*.

[26] Anselme K (2000). Osteoblast adhesion on biomaterials. *Biomaterials*.

[27] Ohgaki M, Kizuki T, Katsura M, Yamashita K (2001). Manipulation of selective cell adhesion and growth by surface charges of electrically polarized hydroxyapatite. *Journal of Biomedical Materials Research*.

[28] Andrade JD, Andrade JD (1985). Principles of protein adsorption. *Surface and Interfacial Aspects of Biomedical Polymers*.

[29] Brash JL, Horbett TA, Brash JL, Horbett TA (1987). Proteins at interfaces: current issues and future prospects. *Proteins at Interfaces: Physicochemical and Biochemical Studies*.

[30] Hing KA (2004). Bone repair in the twenty-first century: biology, chemistry or engineering?. *Philosophical Transactions of the Royal Society A*.

[31] Shimabayashi S, Tamura C, Nakagaki M (1981). Adsorption of hydroxyl ion on hydroxyapatite. *Chemical & Pharmaceutical Bulletin*.

[32] Nishimoto SK, Nishimoto M, Park SW (2008). The effect of titanium surface roughening on protein absorption, cell attachment, and cell spreading. *International Journal of Oral and Maxillofacial Implants*.

[33] Guo CY, Matinlinna JP, Tang ATH (2012). A novel finding of sandblasting’s effect on titanium surface—static charges generation. *Journal of Adhesion Science & Technology*.

